# Species distribution models predict temporal but not spatial variation in forest growth

**DOI:** 10.1002/ece3.2696

**Published:** 2017-03-18

**Authors:** Ernst van der Maaten, Andreas Hamann, Marieke van der Maaten‐Theunissen, Aldo Bergsma, Geerten Hengeveld, Ron van Lammeren, Frits Mohren, Gert‐Jan Nabuurs, Renske Terhürne, Frank Sterck

**Affiliations:** ^1^ Institute of Botany and Landscape EcologyUniversity of GreifswaldGreifswaldGermany; ^2^ Forest Ecology and Forest Management GroupCentre for Ecosystem StudiesWageningen UniversityWageningenThe Netherlands; ^3^ Department of Renewable ResourcesUniversity of AlbertaEdmontonABCanada; ^4^ Laboratory of Geo‐Information Science and Remote SensingWageningen UniversityWageningenThe Netherlands; ^5^ AlterraWageningen University and Research CentreWageningenThe Netherlands

**Keywords:** climate change, dendrochronology, European beech (*Fagus sylvatica*), Norway spruce (*Picea abies*), pedunculate oak (*Quercus robur*), Scots pine (*Pinus sylvestris*), species distribution models, tree growth

## Abstract

Bioclimate envelope models have been widely used to illustrate the discrepancy between current species distributions and their potential habitat under climate change. However, the realism and correct interpretation of such projections has been the subject of considerable discussion. Here, we investigate whether climate suitability predictions correlate to tree growth, measured in permanent inventory plots and inferred from tree‐ring records. We use the ensemble classifier RandomForest and species occurrence data from ~200,000 inventory plots to build species distribution models for four important European forestry species: Norway spruce, Scots pine, European beech, and pedunculate oak. We then correlate climate‐based habitat suitability with volume measurements from ~50‐year‐old stands, available from ~11,000 inventory plots. Secondly, habitat projections based on annual historical climate are compared with ring width from ~300 tree‐ring chronologies. Our working hypothesis is that habitat suitability projections from species distribution models should to some degree be associated with temporal or spatial variation in these growth records. We find that the habitat projections are uncorrelated with spatial growth records (inventory plot data), but they do predict interannual variation in tree‐ring width, with an average correlation of .22. Correlation coefficients for individual chronologies range from values as high as .82 or as low as −.31. We conclude that tree responses to projected climate change are highly site‐specific and that local suitability of a species for reforestation is difficult to predict. That said, projected increase or decrease in climatic suitability may be interpreted as an average expectation of increased or reduced growth over larger geographic scales.

## 1 Introduction

Tree species distributions and forest productivity are strongly linked to climatic factors through direct effects of climate conditions on tree physiological processes (Running et al., 2004). As a consequence, climate change is expected to affect forest growth and health in the short term (Boisvenue & Running, 2006), as well as geographic distribution of species in the long term through demographic processes (Davis & Shaw, 2001; Parmesan & Yohe, 2003). For temperature‐limited boreal forests, the general expectation is a profound northward shift of suitable tree species habitat, while the situation in temperate regions tends to be more complex and varies between Mediterranean, continental, and maritime climates (Bonan, 2008). Direct and indirect climate impacts, such as water shortage and increased risk of forest fires, appear to threaten Mediterranean forests (Allen et al., 2010; Schröter et al., 2005), while forest growth might benefit in continental and Atlantic forests, but only at sites where an increased evaporative demand can be compensated by sufficient water availability (Lindner et al., 2010; Spathelf, van der Maaten, van der Maaten‐Theunissen, Campioli, & Dobrowolska, 2014).

Although predictions of climate change (impacts) still carry uncertainties regarding the magnitude of responses, there is growing awareness among forestry professionals worldwide that climate change potentially poses a large threat to the economic and ecological value of forest lands (Lindner et al., 2014). For Europe, Hanewinkel, Cullmann, Schelhaas, Nabuurs, and Zimmermann (2013) estimated that economic losses may total several hundred billion euros by the end of the century without appropriate adaptation strategies for the forestry sector. To guide species choice in reforestation and forest management prescriptions to address climate change, species distribution models (SDMs) are potentially a useful tool. These correlative models employ a variety of statistical or machine learning techniques to relate species presence/absence data to relevant predictor variables such as climate data, topo‐edaphic variables, or other habitat factors (e.g., Guisan & Zimmermann, 2000). Although there are exceptions (e.g., O'Neill, Hamann, & Wang, 2008), SDMs normally predict the realized niche space of species.

The models have been widely used to illustrate the discrepancy between current tree species distributions and their predicted potential habitat under climate change (e.g., Iverson & Prasad, 1998; Loarie et al., 2008; Thomas et al., 2004; Thuiller, Lavorel, Araújo, Sykes, & Prentice, 2005). The realism and correct interpretation of such projections, however, has been the subject of considerable discussion (e.g., Botkin et al., 2007; García‐Valdés, Zavala, Araújo, & Purves, 2013; Hampe, 2004; Thuiller et al., 2008), and there is consensus in the scientific community that SDMs are conceptually inadequate to accurately predict demographic processes of species under climate change. Modeling the realized climatic niche does not reveal the full range of the species’ ecological tolerances and limitations, and predicted gain or loss of suitable climate habitat does not imply an immediate threat to a species population or rapid expansion of a species’ range.

Despite the limitations of SDMs for climate change impact assessments on complex ecological systems, it has been pointed out that species distribution models are conceptually well suited for simpler practical tasks: guiding climate change adaptation strategies that involve habitat restoration or choosing suitable tree species for reforestation (Gray & Hamann, 2011, 2013; Hamann & Aitken, 2013; Schelhaas et al., 2015). For such management applications, the primary task is to match source and target environments. Nevertheless, it is uncertain whether subsequent long‐term forest growth and forest health are well described by species distribution models that may be used to guide initial decisions on species choice for a general geographic region.

Here, we contribute a retrospective analysis how SDM‐derived habitat suitability projections for four major European tree species (Norway spruce—*Picea abies* (L.) Karst., Scots pine—*Pinus sylvestris* L., European beech—*Fagus sylvatica* L., and pedunculate oak—*Quercus robur* L.) correlate with forest growth data from long‐term inventory plots and tree‐ring chronologies. Our hypothesis is that periods of marginal growth observed in the tree‐ring record (for example, during cold or dry episodes) may be predicted by annual hindcasts of habitat suitability from species distribution models. Such correlations between growth and modeled habitat suitability may vary from site to site. For example, projected habitat loss during drought periods may correlate well with tree‐ring records on water‐limited sites but not on wet sites, which would point to important interactions between climatic and nonclimatic abiotic factors. Notwithstanding large variation from nonclimatic site factors, projections of climatic habitat suitability should to some degree correlate positively with long‐term growth records. Strong spatial (inventory plots) or temporal (tree‐ring‐based) associations of growth with climate may increase our confidence in using species distribution models to guide climate change adaptation strategies in forestry and ecosystem management.

## 2 Methods

### 2.1 Forest inventory and tree‐ring data

We use a forest inventory database for 17 European countries, including Belgium (9,075 plots), Croatia (39), Estonia (1,598), Finland (1,690), France (1,741), Germany (54,087), Italy (13,972), Lithuania (744), the Netherlands (1,442), Norway (8,629), Romania (196), Slovakia (1,410), Slovenia (38), Sweden (2,784), Ukraine (126), and the United Kingdom (19,166). The database was originally compiled by Brus et al. (2012) and Nabuurs (2009). In addition, we added a newly available Spanish forest inventory database (82,527) that is publicly available from the Spanish Ministry of Agriculture, Food and Environment (http://www.magrama.gob.es/es/desarrollo-rural/temas/politica-forestal/inventario-cartografia/inventario-forestal-nacional).

For a subset of 12 countries that still represent a broad range of climate conditions throughout Europe, information on stand age and species‐specific standing stock volumes was available (excluding Croatia, the Netherlands, Romania, Ukraine, and United Kingdom). Rather than attempting age‐based adjustments, we only use volume data for 40‐ to 60‐year‐old stands (hereafter referred to as ~50‐year‐old plot/volume data) resulting in 11,539 plots from a total of 199,264 inventory plots with species‐specific presence/absence data. The presence/absence data were used for building species distribution models that predict habitat suitability, while the subset of ~50‐year‐old plot data was used to analyze associations between habitat suitability and forest growth.

To validate predictions of interannual variation in habitat suitability, we obtained tree‐ring data from 295 sites both from the International Tree‐Ring Data Bank (ITRDB) (NOAA, 2016), as well as from previous studies by van der Maaten (2012) and van der Maaten‐Theunissen, Kahle, & van der Maaten (2013). We detrended all individual tree‐ring series by fitting a cubic smoothing spline with a 50% frequency cutoff at 30 years in order to remove nonclimatic growth responses (see also Cook & Peters, 1981), such as biological age trends or effects of forest management. Indices were calculated dividing the observed by the predicted values, resulting in standardized series that are dimensionless and have a mean of one. Finally, the standardized chronologies for individual trees were averaged per site in so‐called site chronologies using a bi‐weight robust mean.

### 2.2 Climate data

Climate data were generated using the software package ClimateEU (Hamann, Wang, Spittlehouse, & Murdock, 2013; Wang, Hamann, Spittlehouse, & Murdock, 2012), available for anonymous download at http://tinyurl.com/ClimateEU. The ClimateEU package is a software front‐end for interpolated climate databases generated with the Parameter‐elevation Regressions on Independent Slopes Model (PRISM) (Daly et al., 2008). The software allows to query monthly historical climate data from 1901 to 2013, as well as to generate gridded climate surfaces for Europe for habitat suitability modeling. The software implements downscaling algorithms that use empirically derived local lapse rates for individual climate variables to adjust for any discrepancies between the elevation of sample locations (tree‐ring chronologies and inventory plots) and gridded climate databases that the ClimateEU software queries (Hamann et al., 2013; Wang et al., 2012).

We use the 30‐year climate normal period from 1961 to 1990 as a climate reference period, and a 15‐year climate average from 1995 to 2009 to represent recent observed climate change (inventory plot and tree‐ring data were not available for more recent years). We use 10 biologically relevant climate variables that account for most of the variance in climate data while avoiding multicollinearity: mean annual temperature, the mean temperatures of the warmest and coldest month, the difference between July and January temperature as an indicator of continentality, mean annual precipitation, May to September (growing season) precipitation, growing degree days above 5°C, frost‐free days, and two dryness indices after Hogg (1997): an annual climate moisture index and a June–August summer climate moisture index. The variables are explained in detail by Wang et al. (2012).

For spatial habitat modeling, we use 1‐km resolution climate grids in Albers Equal Area projection. Species distribution models were built based on a widely used reference period that largely predates a significant anthropogenic warming signal (the 1961–1990 climate normal). Projections were made for this reference normal period, a more recent average (1995–2009), as well as for the 2020s (2011–2040) and 2050s (2041–2070) using an ensemble average from the CMIP3 multimodel dataset corresponding to the fourth IPCC assessment report (Meehl et al., 2007). Similar to Fordham, Wigley, & Brook (2011), we excluded poorly validated AOGCMs (MIROC3.2, MRI‐CGCM2.3.2, MIROC3.2, IPSL‐CM4, FGOALS‐g1.0, GISS‐ER, GISS‐EH, and GISS‐AOM) and retained the remaining CMIP3 models. The study was initiated before the CMIP5 dataset corresponding to the fifth IPCC assessment report became available. However, we note that the two AOGCM generations yield remarkably similar projections in magnitude, uncertainty, and spatial resolution (Knutti & Sedláček, 2013). Accounting for different approaches to describe emission scenarios (SRES versus RCP), we find that the projections at the level of multimodel ensemble averages remain largely the same for both temperature and precipitation variables.

### 2.3 Species distribution modeling

We built species distributions using the RandomForest ensemble classifier (Breiman, 2001) implemented by the randomForest package (Liaw & Wiener, 2002) for the R programing environment (R Development Core Team, 2016). This ensemble classifier grows multiple classification trees (here, *n* = 500) from bootstrapped samples of the training data and determines its prediction by majority vote over all developed classification trees (Cutler et al., 2007). Importance values for the predictor variables were calculated as the frequency that a particular climate variable contributed to a correct classification. Habitat projections were made (1) for climate data for the 1961–1990 period to analyze associations between projected habitat suitability and growth observed on forest inventory plots, (2) for climate data for individual years from 1901 to 2009 to analyze associations between projected habitat suitability and growth tree‐ring width, and (3) for a recent climate average (1995–2009) and future periods (2020s and 2050s) to infer future trends in growth patterns across Europe.

The RandomForest algorithm was preferred over other algorithms, like maxent, because we had a rather unproblematic dataset with a high number of census records. To account for nonlinearity in the species response across climate gradients and for interactions among climate variables, RandomForest is generally considered the most powerful implementation of regression tree techniques.

We also made an attempt to use the regression tree (rather than classification tree) functionality of RandomForest to model a continuous response variable (i.e., ~50‐year‐old plot volume data) as a function of climate. For the volume models, we built, for each of the studied tree species, a training dataset of approximately 9,000 samples, which were climatically characterized and comprised equal numbers of plots with volume data, absence plots, and random absences. Random absences were only selected for countries without plot‐data availability by using an overlay with tree species distributions from the European Forest Genetic Resources Programme (EUFORGEN, 2012). We removed pseudo‐absences using a *p *> .5 threshold for species presence using RandomForest habitat projections based on presence/absence variants of the training datasets. This analysis did not yield acceptable validation statistics, and we briefly report on this negative result for the inventory data‐climate modeling attempt.

Model performance was evaluated using the area under the receiver operating characteristic curve (AUC of ROC) to evaluate the statistical accuracy of the species distribution models for individual tree species. The AUC statistic is a common measure of the performance of classification rules; it balances the ability of a model to detect a species when it is present (sensitivity) against its ability to not predict a species when it is absent (specificity) (e.g., Fawcett, 2006; Fielding & Bell, 1997). We further report model sensitivity (calculated as TP/(TP + FN) with TP as true positives and FN as false negatives) and model specificity (TN/(TN + FP) with TN as true negatives and FP as false positives). All ROC and AUC calculations were implemented with the ROCR package (Sing, Sander, Beerenwinkel, & Lengauer, 2005) for the R programming environment.

### 2.4 Statistical analysis

To assess whether habitat projections of our species distribution models are associated with observed forest growth, we conducted a Pearson correlation analysis between the probability of presence estimates from the RandomForest‐based habitat suitability projections and growth measurements from two data sources: inventory plots (to represent spatial variation) or growth increments from tree‐ring data (to represent temporal variation). All correlations were visually checked for linearity, and transformations were judged as unlikely to have any notable or consistent effect on the results. Habitat projections for correlations with ~50‐year‐old plot volume data were based on one long‐term projection for the 1961–1990 normal period (to analyze spatial growth−climate associations). Habitat projections for comparison with tree‐ring increments were based on habitat projections for 109 individual years, from 1901 to 2009 (to analyze temporal growth−climate associations).

Because tree‐ring chronologies are known to display temporal autocorrelations (Fritts, 1976), we allowed for a maximum lag of 3 years for the correlation analysis between annual habitat projections and site chronologies. Further, because forest ecosystems can be well buffered against short‐term climate fluctuations, we also evaluated correlations based on 3‐, 5‐, 7‐, and 9‐year moving averages of both habitat projections and site chronologies. Generally, 5‐year moving averages generated the strongest correlation coefficients (both negative and positive), and was therefore applied to all chronologies. To determine whether the average correlation coefficient was larger than zero, a single‐sample one‐tailed t‐test across all correlation coefficients from each species was carried out, testing to our overall scientific null hypothesis that there was no positive association between growth and climatic habitat suitability.

## 3 Results

### 3.1 Variable importance and model statistics

RandomForest importance values indicate that climate predictors of species ranges are quite species‐specific, with a notable contrast between coniferous and deciduous trees (Table 1). For spruce and pine, the mean warmest month temperature contributes strongly to correctly predicting presence and absence of these species in inventory plot data. Further, the summer and annual climate moisture indices were important predictor variables for spruce. In contrast, variable importance values for summer heat and moisture variables were very low for the broadleaved tree species oak and beech. For these species, continentality and other cold‐related variables stand out as the best predictors of species ranges. Continentality is the most important predictor for both broadleaves, with mean coldest month temperature the second most important predictor for beech occurrences and several variables, including frost‐free period, being secondary predictors for oak.

**Table 1:** Importance values of RandomForest climate predictor variables, calculated as the number of times that a particular variable contributed to a correct classification. Reported values are divided by 100 for readability

Climate variable	RF importance			
	Norway spruce	Scots pine	European beech	Pedunc. oak
Mean annual temperature (°C)	2.8	2.0	1.8	1.7
Mean warmest month temperature (°C)	7.8	8.9	3.2	3.0
Mean coldest month temperature (°C)	5.6	2.0	4.9	2.3
Continentality (°C)	4.9	4.1	8.0	4.6
Mean annual precipitation sum (mm)	3.1	2.5	3.2	1.2
Growing season precipitation sum (mm)	4.3	4.3	2.2	1.9
Growing degree days >5°C (days)	5.8	6.0	2.4	2.8
Frost‐free period (days)	4.6	2.0	2.6	3.0
Annual climate moisture index (cm)	6.7	5.1	2.0	2.6
June–August climate moisture index (mm)	8.5	4.9	2.1	2.8

Accuracy statistics for the presence/absence predictions are shown in Table 2. Total error rates of false positives and false negatives range between 0.13 and 0.32, with the widespread coniferous species having the highest error rates. AUC values are fairly high ranging from 0.72 to 0.90, with beech and oak having the best predictive accuracy. For all species, the number of false‐negative errors is higher than the number of false‐positive errors, which indicates that model prediction errors are mainly driven by falsely predicting species absence. Similarly, model sensitivity is low and model specificity is high, showing that true species absences were well modeled.

**Table 2:** Predictive accuracy statistics for the projected distribution areas of the four study species

Species	Error rate	Specificity	Sensitivity	AUC
Norway spruce	0.25	0.71	0.65	0.81
Scots pine	0.32	0.63	0.60	0.72
European beech	0.16	0.85	0.62	0.90
Pedunculate oak	0.13	0.79	0.70	0.90

Error rate = (False Positive + False Negative)/(Total Positive + Total Negative).

**Figure 1:**
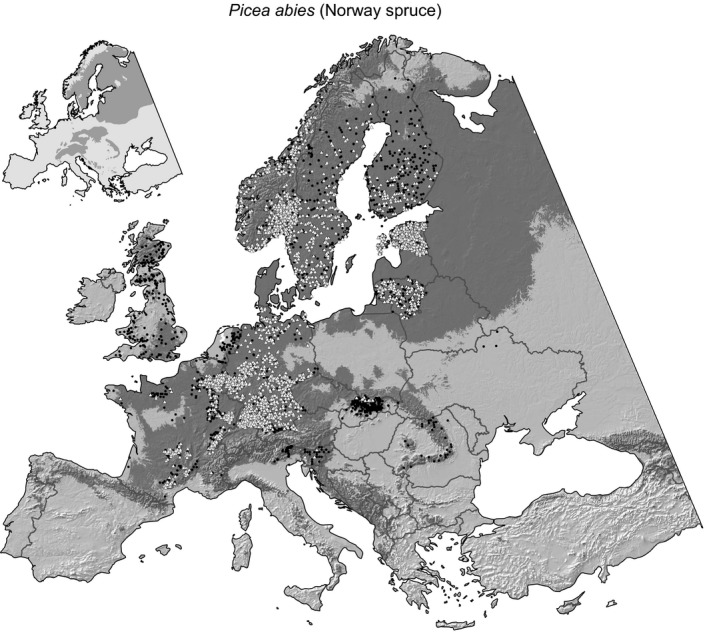
Sample plot data for the Norway spruce presence (●) and inventory plots that also contained height, diameter, and volume data (Δ). The modeled species distribution is based on probability of presence estimate above .4 (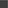), where false‐positive and false‐negative presence/absence predictions are minimized (see Table 2 for statistics). Note that absence data were omitted from the figure. The inset shows the approximate natural distribution of the species according to EUFORGEN (2012)

The results of the distribution model for Norway spruce are shown in Figure 1, with corresponding maps for Scots pine, European beech, and pedunculate oak provided as Supporting information (Figs S1–S3). A comparison of both the presence data and the predicted species distribution with the approximate natural distribution (after EUFORGEN, 2012; see inset Figure 1) reveals substantial differences. Today's distribution of spruce across Europe has a distinctively wider range than its natural distribution suggests, due to Norway spruce being widely planted as a highly valued forestry species for timber production in Europe.

### 3.2 Habitat projections versus growth

**Figure 2:**
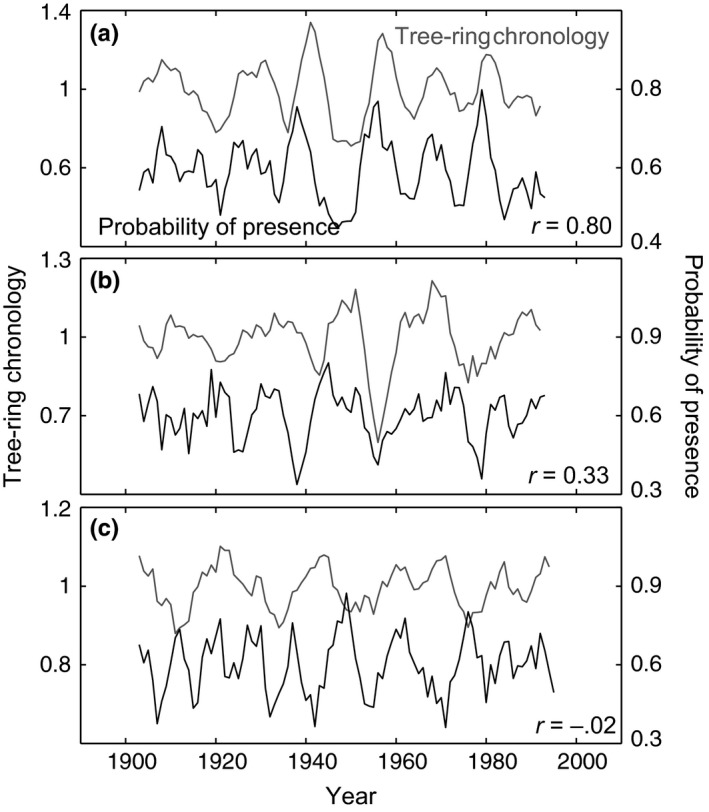
Time series of tree‐ring indices and predictions of habitat suitability for three sample chronologies of Norway spruce with a high (a), a low (b), and a negative (c) correlation coefficient. For a map of Norway spruce correlation coefficients see Figure 3. For distribution statistics of all correlation coefficients for all species, see Table 3

**Figure 3:**
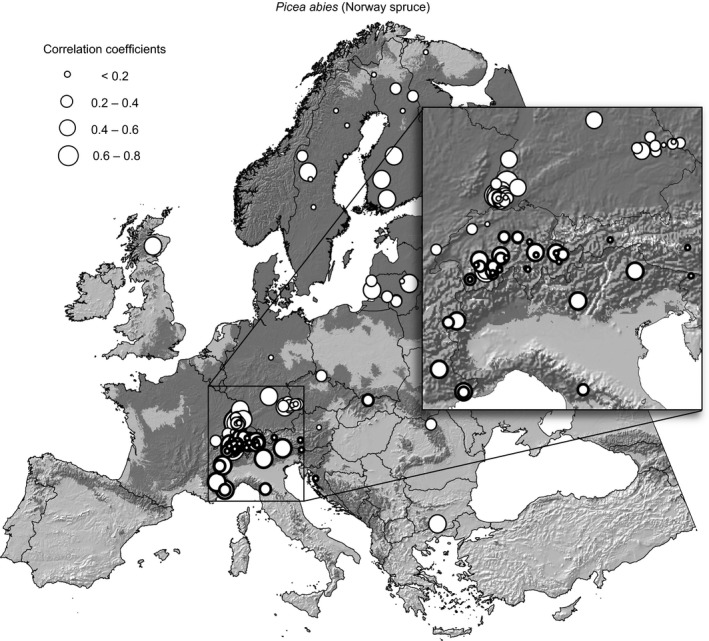
Correlation coefficients between predictions of the Norway spruce species distribution model for 5‐year moving averages of habitat suitability hindcasts (1901–2009), and corresponding 5‐year moving averages of site chronologies. The location of site chronologies is indicated by circles, the size of circles represents the strength of the correlation, and chronologies from sites higher than 1,500 m are marked by thick borders

The analysis of temporal growth–climate associations based on habitat suitability predictions and annual growth increments in 295 tree‐ring chronologies reveals that these correlations are highly variable in magnitude and even direction. Overall, we found an average correlation across all four species of .22 but with correlation coefficients from individual chronologies as high as .82, often approaching zero, and regularly being negative with values up to −.31 (Table 3). Examples for time series with positive, neutral, and negative associations are shown in Figure 2, and a map of the resulting correlation coefficients for all individual chronologies of Norway spruce is shown in Figure 3. No spatial patterns are apparent, and elevation or climate normal variables for the origin of sample locations were not associated with the strength or directions of correlation coefficients (data not shown).

**Table 3:** Results of Pearson correlation analyses between site chronologies of tree‐ring data and annual habitat suitability hindcasts

Species	*N*	Distribution of correlation coefficients (*r*)			*p* (*r *≤* *0)
		Minimum	Mean	Maximum	
Norway spruce	126	−.31	.25	.82	<.0001
Scots pine	128	−.27	.18	.61	<.0001
European beech	4	−.10	.31	.49	.1083
Pedunculate oak	37	−.22	.19	.72	<.0001

Correlation coefficients (*r*) were calculated between 5‐year moving averages of the habitat hindcasts (1901–2009), and corresponding 5‐year moving averages of site chronologies. The number of chronologies (*N*), the minimum, mean, and maximum *r*, as well as the probability that the mean correlation coefficient for each species is smaller or equal to zero, are reported.

Even though there are no apparent associations of correlation coefficients with geographic or climatic variables of the sample locations, the average association between climate suitability and growth inferred from ring width is positive and overall highly significant. The probability of the true average correlation having a value of zero or smaller (one‐sided *t*‐test) is negligible, except for European beech, where we lack the sample size to reject the null hypothesis with confidence (Table 3).

Habitat projections for inventory plot locations based on the 30‐year climate normal period (1961–1990) for each location were not significantly correlated with standing volume at age ~50. In fact, visual examinations suggest no associations at all with correlation coefficients approaching zero for all species (data not shown). Similarly, correlations between direct RandomForest model predictions of standing volume trained with ~50‐year‐old plot volume data and validated against a withheld dataset of plot measurements approach zero (data not shown).

### 3.3 Future habitat suitability projections

**Figure 4:**
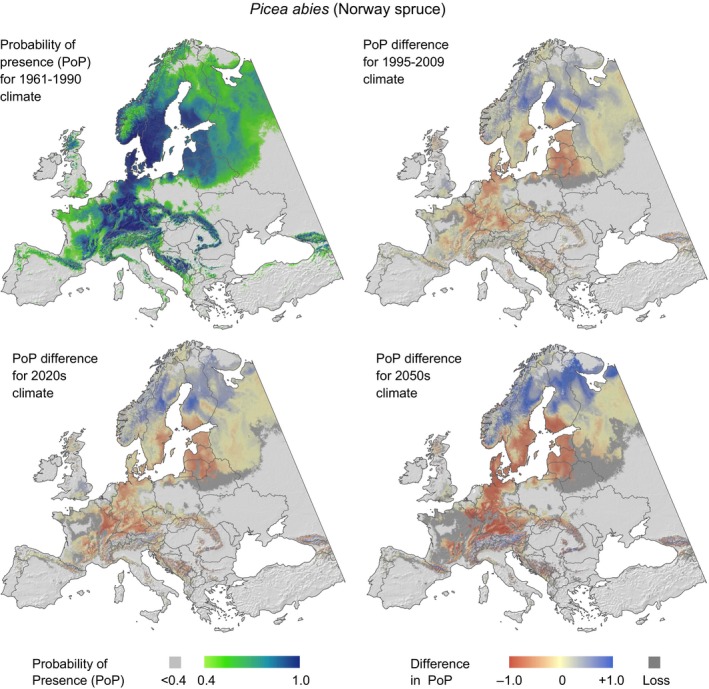
Predicted climatic habitat suitability for Norway spruce based on the climate period of the training dataset (1961–1990), and changes in habitat suitability for a recent 15‐year climate period (1995–2009), and ensemble projections for the 2020s and 2050s of the CMIP3 multimodel dataset for the emission scenario A2. Note that predictions for all climate periods have been limited to the current extent of the species range. If the absolute probability of presence was predicted to be below .4, the habitat is marked as lost relative to the 1961–1990 baseline projection

The above analysis shows that model outputs must be interpreted with caution. Our validations against growth data from tree rings and inventory plots suggest that habitat projections are not informative at a forest stand level and only represent average expectations over larger geographic areas. We remind the reader that stand level variation in effects of climate change on tree growth may be very large (and occasionally reversed) when compared to the general expectation of growth trends under climate change. With these caveats clearly stated, Figure 4 illustrates changes in modeled habitat suitability and by inference also expected changes in tree growth for Norway spruce under recently observed climate change (1995–2009) and future climate periods (2020s and 2050s). Figures S4–S6 show corresponding projections for Scots pine, European beech, and pedunculate oak.

Within the current extent of the species range, habitat suitability of spruce generally decreases in southern and central Europe, whereas it increases (or remains stable) in the north or at higher elevation. Similar trends are observed for pine, beech, and oak (Figs S4–S6). Further, our model projections indicate that climate change is happening as fast as or faster than projected by general circulation models. Namely, hindcast projections for 1995–2009 period already appear approximately equal to 2020s projections.

## 4 Discussion

### 4.1 Species distribution model evaluation

Our species distribution models appear to be reasonably accurate when validated against withheld presence/absence data. The two broadleaved tree species (oak and beech) had very good validation statistics with AUCs of .9, while the two conifers (pine and spruce) had moderate predictive accuracies with AUCs between .7 and .8. The importance values of predictor variables appear largely sensible: The coniferous species had no range limitations toward cold northern climates and their ranges within the study area were therefore best predicted by warm temperatures and dryness, delineating the southern and low‐elevation range limits (Table 1, Figures 1 and S1). In contrast, the species range of the two broadleaved trees were bound toward northern and high‐elevation range limits within the study area as well. Therefore, a broader range of climate variables had predictive value (Table 1, Figs S2 and S3).

Notably, when comparing the insets of Figure 1 and the corresponding Figs S1–S3, which reflect the original native species ranges (EUFORGEN 2016) with the modeled species ranges and the occurrence records (shown in the main map of the same figures), it appears that the species distribution model generally overpredicts. However, tree species in Europe have been planted outside of their natural range for centuries (Lindner et al., 2014), and the species distribution model therefore likely reflects the larger fundamental niche of the species. While the fundamental niche (i.e., the absolute environmental tolerances) cannot be comprehensively inferred from plot data (because we do not know which environments were never tested), our modeling effort might approach the species’ fundamental niche (because of planting efforts outside the natural range). Habitat loss projected by the models in this study (putatively approaching the fundamental niche) should therefore be of higher concern than projections of a more restricted realized niche model (based on a just the natural species’ range). Habitat loss in this study could imply that projected climates are outside the species tolerances rather than just favoring competitors in the long term.

### 4.2 SDM projections versus growth records

In correlating model outputs of habitat suitability with growth records from tree rings, all species showed a very similar range of correlation coefficients (Table 3), which reflects a similarly balanced set of projections of changes (compare Figures 4 and S4–S6). The species do not differ dramatically in their sensitivity to climate change based on continental‐scale projections, nor do they differ dramatically in their response to interannual climate variation at the specific sites where they occur based on tree‐ring chronologies. Further, the range of correlation coefficients is surprisingly large and includes negative as well as positive associations with climate suitability projections. This result may not be unexpected if the analysis was carried out for individual climate variables. For example, a high‐temperature anomaly would be expected to yield good growth at the northern edge of the species distribution but lower than average performance at the southern range limit. This is, however, not a plausible explanation for the result at hand. RandomForest is a regression tree classifier and therefore well capable of modeling nonlinear growth responses to predictor variables, as well as interactions among those variables. The negative result further conforms to Figure 3 which does not show any spatial patterns that are associated with the strength of the correlations.

We conclude that tree responses to projected climate change are highly site‐specific and that local suitability of a species for reforestation is difficult to predict. This is further confirmed by our unsuccessful attempt to predict standing volume at an age of ~50 years directly with a RandomForest classifier. RandomForest projections explained less than 5% of the overall variance in this continuous predictor variable, indicating that local site factors overwhelm climate variables as predictors. It should be noted, however, that this applies to sites where the species occurs and has been growing ~50 years. Thus, the inference that climate has no influence on tree growth should not be made from this observation. Rather, we conclude that for well‐established trees at local sites, the cumulative volume over a period of 50 years is not a function of climate but predominantly a function of other site factors.

### 4.3 Regional‐scale growth projections

Despite the wide range of correlation coefficients between habitat suitability projections and tree‐ring records (Table 3), we have strong indications that they do not represent random noise, but real climate habitat–growth associations. The correlation coefficients are generally positive with an average of .22 and differ highly significantly from zero. The probability to obtain an equal or larger average correlation coefficient by random chance is extremely small for all species, except for European beech, where we were only able to evaluate four tree‐ring chronologies. Thus, while local site factors such as soils, topographic exposure, and ground water availability may account for how climate change may affect forest growth at individual sites, significant positive correlations across multiple sites (Table 2) suggest that habitat projections are capable of correctly predicting growth trends on average. Therefore, we conclude that SDM projections should be interpreted as average expectations of increased or reduced growth over larger geographic scales.

Model predictions for the 1995–2009 climate period compared to the 1961–1990 baseline, representing a 28‐year warming trend (i.e., midpoint 2002 minus midpoint 1975), reveal that habitat suitability of Norway spruce declined in the more southern and drier parts of its current distribution, whereas it increased in the north (Figure 4). Interestingly, projections driven by already observed climate change very closely resemble the 2020s projection. This implies that climate change appears to materialize faster than projected even under the pessimistic A2 SRES scenario, which is the basis for the revised RCP 8.5 scenario, that is, an emission storyline that assumes high population growth and lower incomes in developing countries (van Vuuren et al., 2011). While this outlook should raise concern, we show in this paper that the climate impacts on forest growth will be highly variable at local scales. As a consequence, climate change adaptation strategies for the forestry sector that aim at moving species to appropriate local site conditions appear at least as promising as large‐scale geographic assisted migration efforts.

## Conflict of Interest

None declared.
