# Supplementary material for: Species distribution models predict temporal but not spatial variation in forest growth
Source: Ecol Evol. 2017 Mar 18;7(8):2585–94. doi: 10.1002/ece3.2696 (PMC5395440; doi:10.1002/ece3.2696)
Supplement: Supplementary file 1 [file ECE3-7-2585-s001.pdf]

Pinus sylvestris (Scots pine)

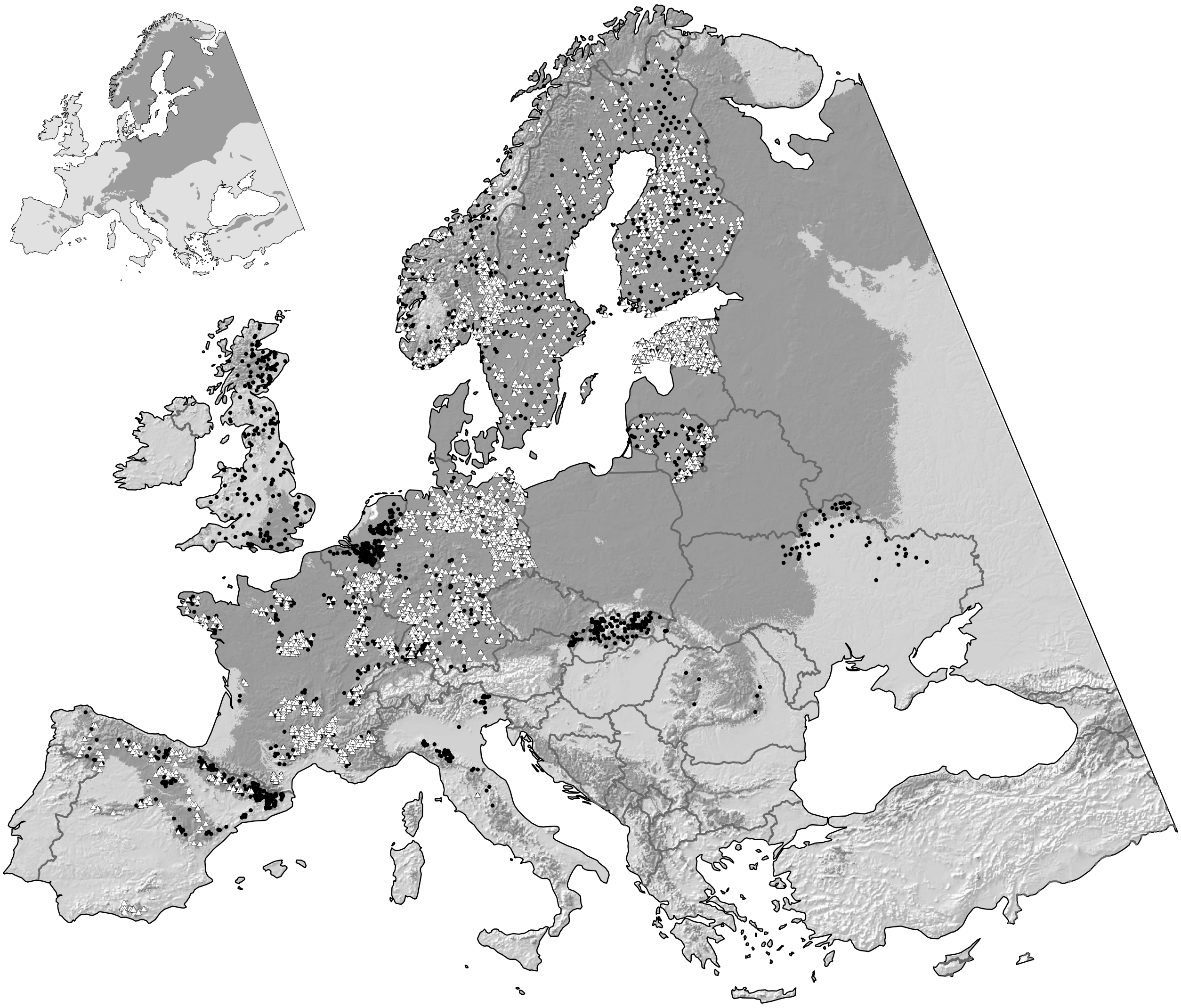

Figure S1. Sample plot data for the Scots pine presence (●), and inventory plots that also contained height, diameter, and volume data (△). The modeled species distribution is based on probability of presence estimate above 0.36 (■), where false positive and false negative presence-absence predictions are minimized (see Table 2 for statistics). Note that absence data were omitted from the figure. The inset shows the approximate natural distribution of the species according to EUFORGEN (2012).

Fagus sylvatica (European Beech)

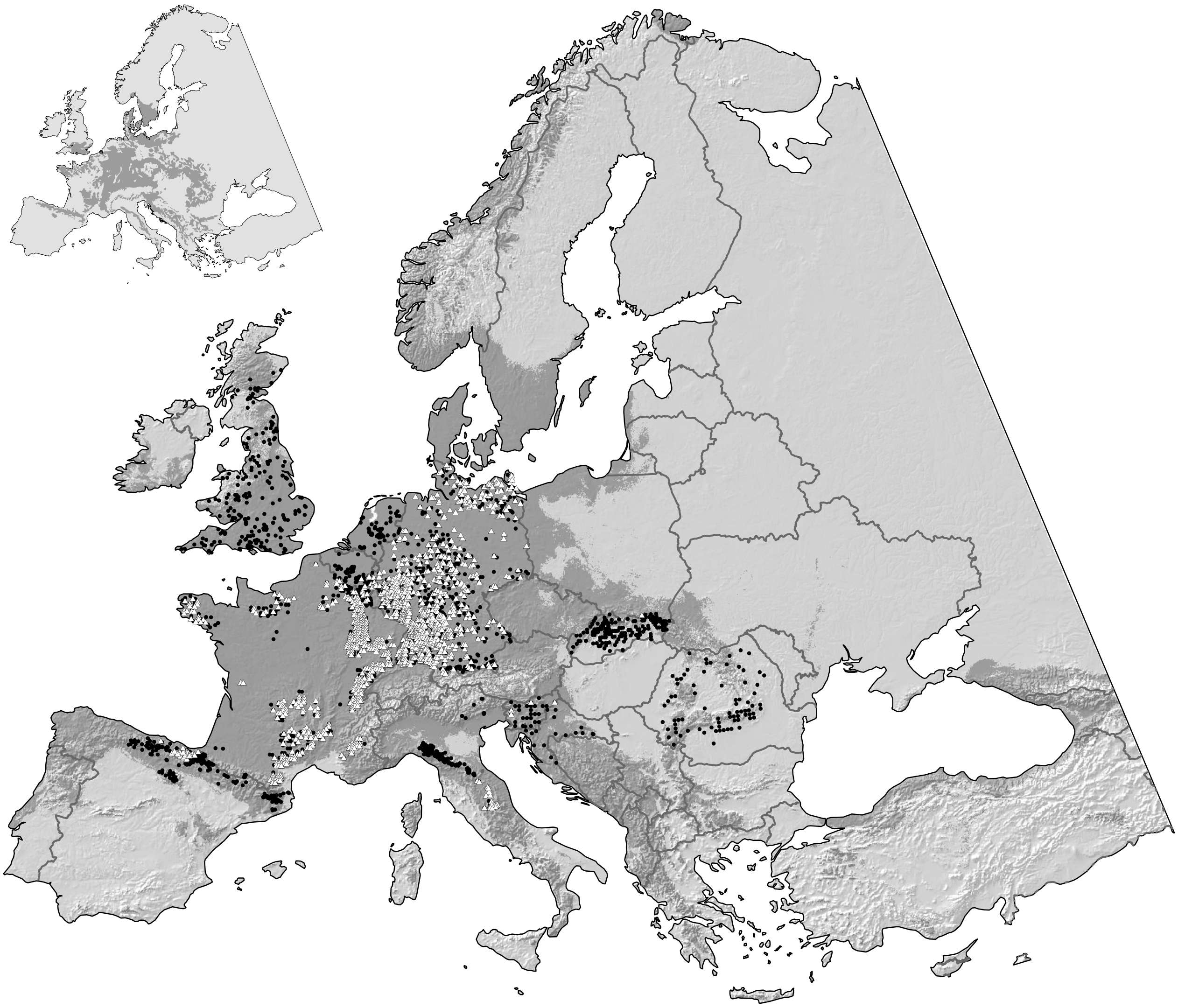

Figure S2. Sample plot data for the European beech presence (●), and inventory plots that also contained height, diameter, and volume data (△). The modeled species distribution is based on probability of presence estimate above 0.35 (■), where false positive and false negative presence-absence predictions are minimized (see Table 2 for statistics). Note that absence data were omitted from the figure. The inset shows the approximate natural distribution of the species according to EUFORGEN (2012).

Quercus robur (pedunculate oak)

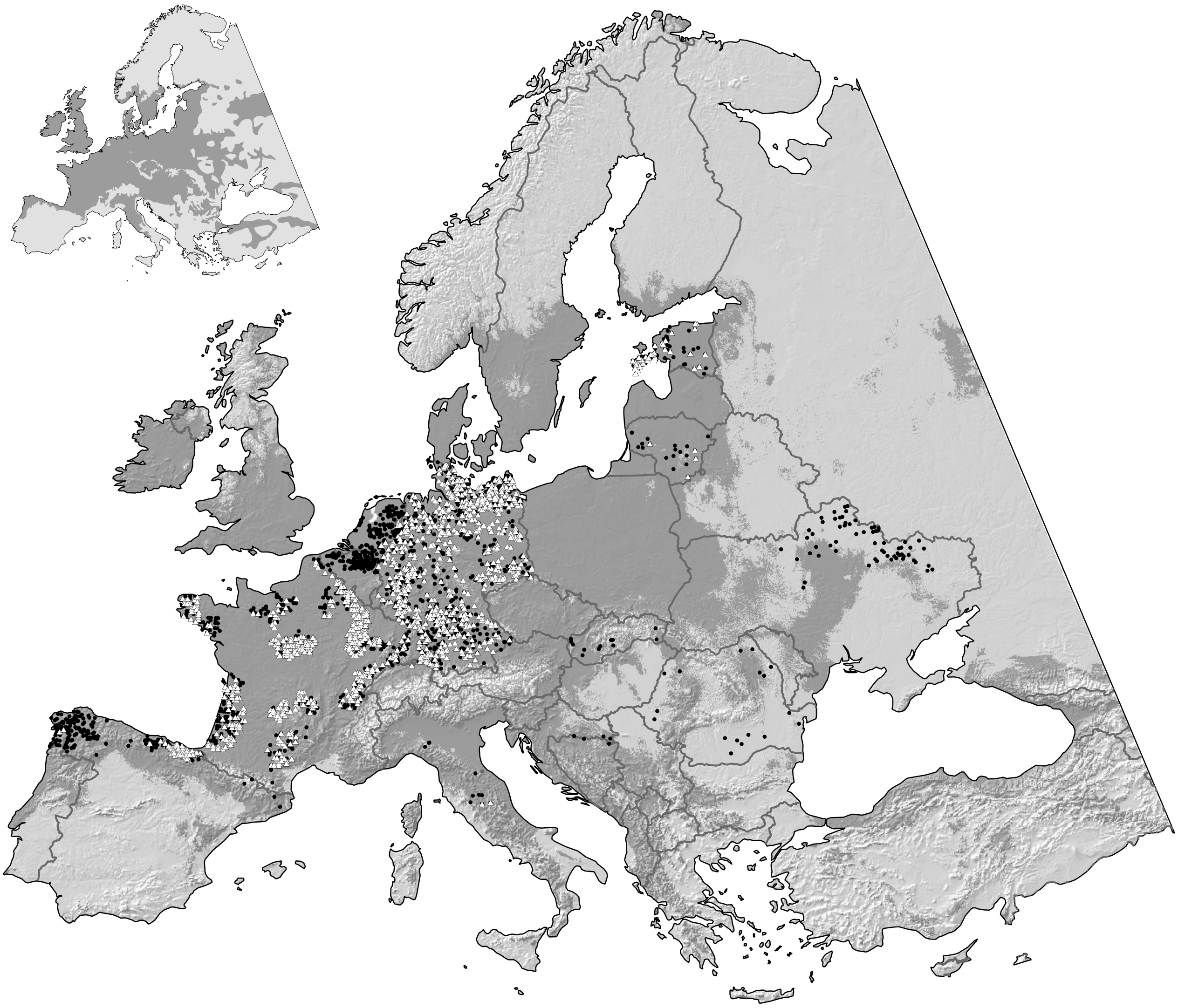

Figure S3. Sample plot data for the pedunculate oak presence (●), and inventory plots that also contained height, diameter, and volume data (△). The modeled species distribution is based on probability of presence estimate above 0.39 (■), where false positive and false negative presence-absence predictions are minimized (see Table 2 for statistics). Note that absence data were omitted from the figure. The inset shows the approximate natural distribution of the species according to EUFORGEN (2012).

Pinus sylvestris (Scots pine)

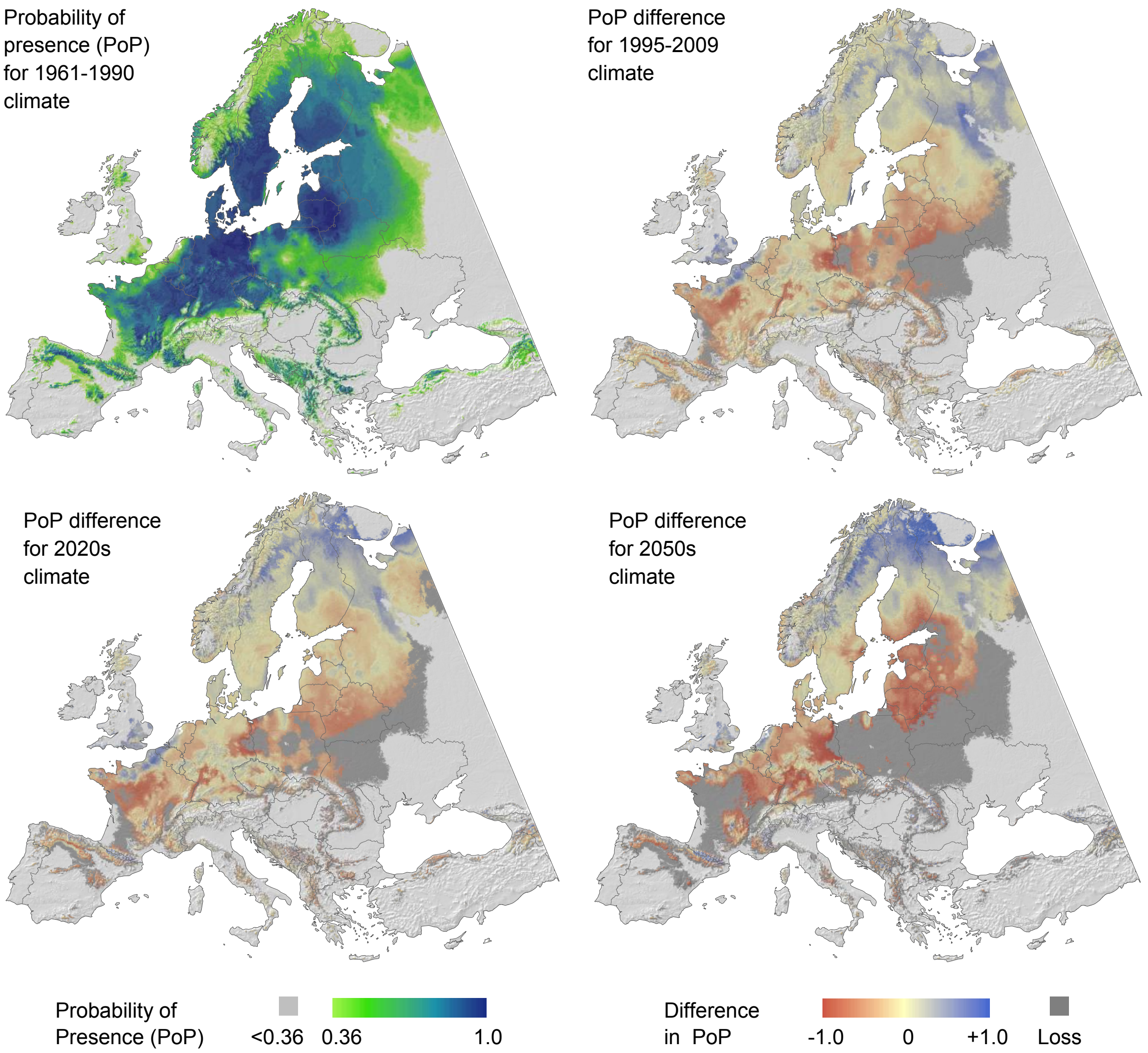

Figure S4. Predicted climatic habitat suitability for Scots pine based on the climate period of the training dataset (1961-1990), and changes in habitat suitability for a recent 15-year climate period (1995-2009), and ensemble projections for the 2020s and 2050s of the CMIP3 multimodel dataset for the emission scenario A2. Note that predictions for all climate periods have been limited to the current extent of the species range. If the absolute probability of presence was predicted to be below 0.36, the habitat is marked as lost relative to the 1961-1990 baseline projection.

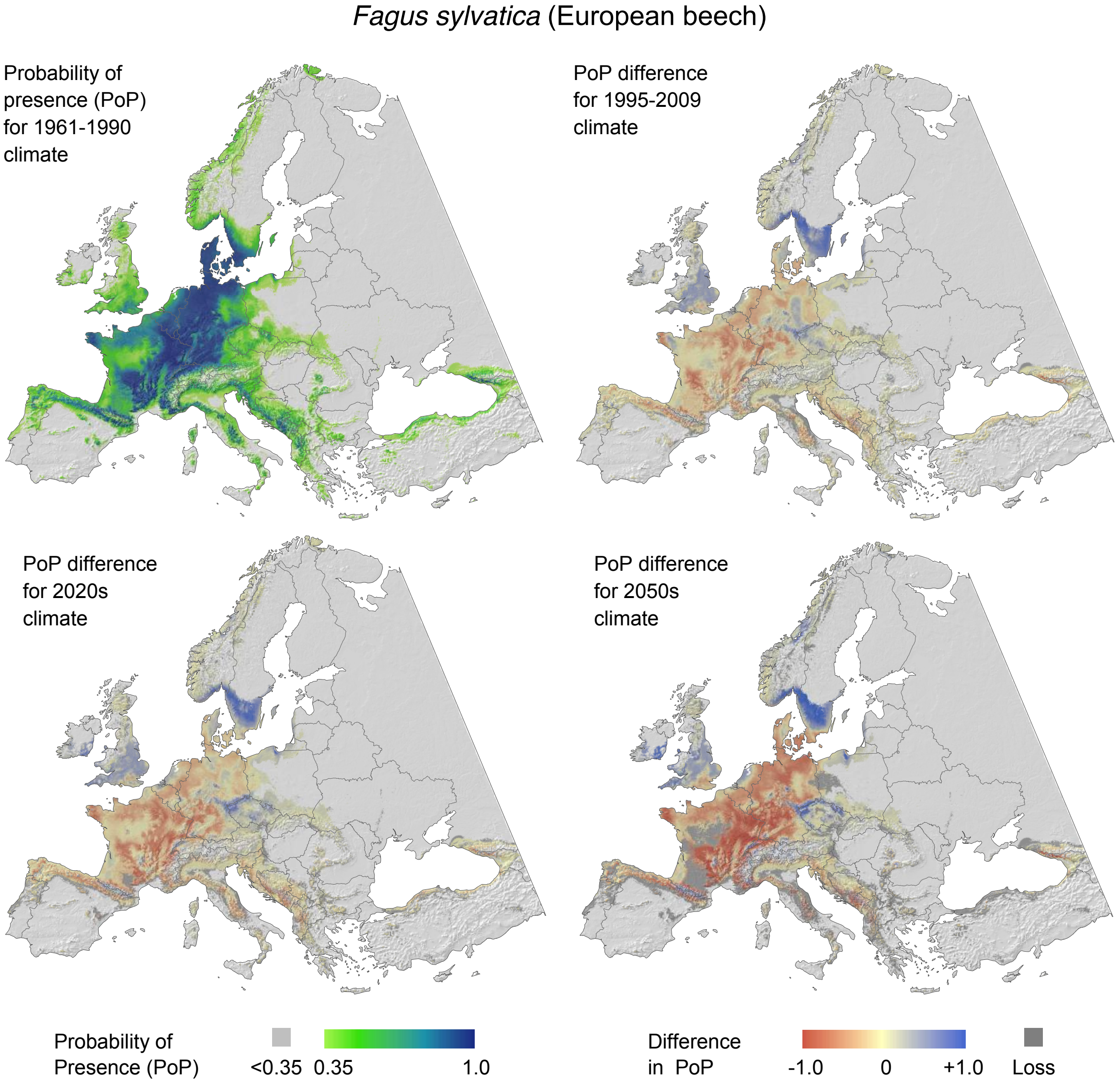

Figure S5. Predicted climatic habitat suitability for European beech based on the climate period of the training dataset (1961-1990), and changes in habitat suitability for a recent 15-year climate period (1995-2009), and ensemble projections for the 2020s and 2050s of the CMIP3 multimodel dataset for the emission scenario A2. Note that predictions for all climate periods have been limited to the current extent of the species range. If the absolute probability of presence was predicted to be below 0.35, the habitat is marked as lost relative to the 1961-1990 baseline projection.

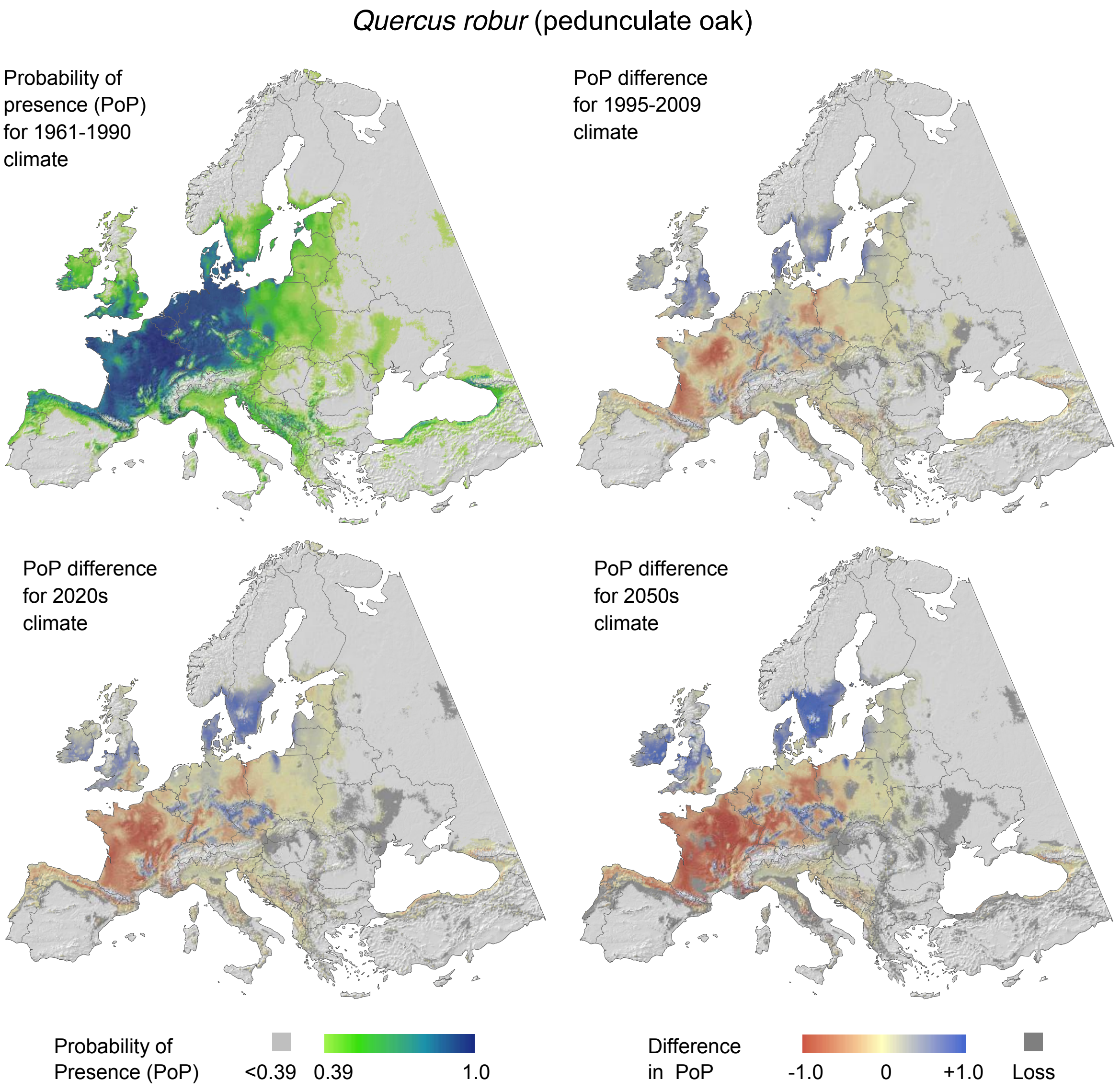

Figure S6. Predicted climatic habitat suitability for pedunculate oak based on the climate period of the training dataset (1961-1990), and changes in habitat suitability for a recent 15-year climate period (1995-2009), and ensemble projections for the 2020s and 2050s of the CMIP3 multimodel dataset for the emission scenario A2. Note that predictions for all climate periods have been limited to the current extent of the species range. If the absolute probability of presence was predicted to be below 0.39, the habitat is marked as lost relative to the 1961-1990 baseline projection.
